# Demographic and Indication-Specific Characteristics Have Limited Association With Social Network Engagement: Evidence From 24,954 Members of Four Health Care Support Groups

**DOI:** 10.2196/jmir.6330

**Published:** 2017-02-17

**Authors:** Trevor van Mierlo, Xinlong Li, Douglas Hyatt, Andrew T Ching

**Affiliations:** ^1^ Research Associate Henley Business School University of Reading Henley-on-Thames United Kingdom; ^2^ Evolution Health Systems Inc Toronto, ON Canada; ^3^ Rotman School of Managment University of Toronto Toronto, ON Canada

**Keywords:** econometric models, social networking, social support, self-help groups, data mining, Internet, regression analysis, forecasting, superusers

## Abstract

**Background:**

Digital health social networks (DHSNs) are widespread, and the consensus is that they contribute to wellness by offering social support and knowledge sharing. The success of a DHSN is based on the number of participants and their consistent creation of externalities through the generation of new content. To promote network growth, it would be helpful to identify characteristics of superusers or actors who create value by generating positive network externalities.

**Objective:**

The aim of the study was to investigate the feasibility of developing predictive models that identify potential superusers in real time. This study examined associations between posting behavior, 4 demographic variables, and 20 indication-specific variables.

**Methods:**

Data were extracted from the custom structured query language (SQL) databases of 4 digital health behavior change interventions with DHSNs. Of these, 2 were designed to assist in the treatment of addictions (problem drinking and smoking cessation), and 2 for mental health (depressive disorder, panic disorder). To analyze posting behavior, 10 models were developed, and negative binomial regressions were conducted to examine associations between number of posts, and demographic and indication-specific variables.

**Results:**

The DHSNs varied in number of days active (3658-5210), number of registrants (5049-52,396), number of actors (1085-8452), and number of posts (16,231-521,997). In the sample, all 10 models had low R^2^ values (.013-.086) with limited statistically significant demographic and indication-specific variables.

**Conclusions:**

Very few variables were associated with social network engagement. Although some variables were statistically significant, they did not appear to be practically significant. Based on the large number of study participants, variation in DHSN theme, and extensive time-period, we did not find strong evidence that demographic characteristics or indication severity sufficiently explain the variability in number of posts per actor. Researchers should investigate alternative models that identify superusers or other individuals who create social network externalities.

## Introduction

### Background

Digital health social networks (DHSNs), otherwise known as discussion forums or peer-to-peer support groups, are in abundance [1-8]. Although the efficacy of these networks is still being evaluated, the consensus is that social support and knowledge sharing increase patient education, enhance self-management, and decrease burden on existing health services [9-16].

In an era of increasing health costs [17,18], an aging population [19-22], and an annual US $300 billion adherence problem [23-26], DHSNs are beginning to play an important role in improving the delivery of North American health services [27,28].

As we increasingly rely on technology to help us look after our health, management science is playing a greater role in using data to measure efficiencies [29-31]. In the case of DHSNs, analysis is now turning to mechanisms that drive growth, help attain sustainability, and generate positive network externalities.

### Research on Social Network Structure, Growth, and Sustainability

As a discipline, social network theory (SNT) maps social capital and the strength of relationships in networks. Within a network, nodes are individual actors, and ties are the relationships between nodes. For decades, disciplines such as economics, political science, public health, marketing, and finance have analyzed real world relationships within networks of actors [32-37]. These studies typically leverage graph theory, sociograms, or stochastic models to examine relationships [38-40].

Recently, SNT has shifted toward the topology of scale-free networks. This stream of research investigates whether network growth is random, if networks evolve, follow encoded and organized principles [41-46], and if taxonomies of actors naturally exist [47-51].

### Three Fundamentals of Digital Health Social Networks

In the context of this study, actors are DHSN registrants who have created, at minimum, 1 post. From this perspective, 3 fundamental principles guide network growth.

The first is the network’s total number of posts. In most DHSNs, actor posts remain on the network, and each new post adds to the quantitative size and value of the community. Whether actors passively read, actively respond to, or agree or disagree with new content, the quantitative value of the network *n* increased with each new post by *n* +1. In management and economics literature this is referred to as positive network externalities [52].

Second is the number of actors in the network. If a network contains *x* actors, potential connections between actors is *x* (*x*−1). The greater the number of actors, the greater the potential for network expansion and the generation of new externalities. This has been illustrated in the study of networks in demand-side economies, where the value of a product or service is directly related to the number of others who use it [53,54].

Third, the mathematical relationship between these 2 quantities (positive network externalities and number of actors) represents a power law [55-57]. Marketing experts have observed this phenomenon and have intuitively referred to it as the 1% rule or the 90-9-1 principle [58,59]. Both concepts are related to the Pareto principle [60], and applied empirically, they have shown to be intrinsic to social network structure [61-63].

Monitoring nodes and ties, and monitoring topologies are important considerations for those who manage social networks. However, these tasks are retrospective as they examine a network’s past state. Methods to drive future growth and promote individual agency are required. As the creation of externalities governs the success of a network, it would be helpful to profile actors who create value by generating externalities [64].

### The Interventions

The 4 interventions in this study [65-68] contained self-guided interactive behavior change treatment programs based on state-of-the-art best practice, and have been examined extensively in the literature [69-83]. A component of each of the interventions is a DHSN moderated by trained and paid employees. All posts are reviewed and approved by a moderator, and any post that does not address the indication is permanently removed. Moderators can also instantaneously communicate with all actors. Table 1 outlines each program’s theoretical constructs and evidence base.

**Table 1:** Theoretical constructs and evidence-base.

Theoretical construct	Problem drinking	Depressive disorder	Panic disorder	Smoking cessation
Brief intervention [84]	X	X	X	X
Cognitive behavioral therapy [85]		X	X	
Gamification [86]	X	X	X	X
Harm reduction [87]	X			X
Health belief model [88]	X	X	X	X
Motivational interviewing [89]	X	X	X	X
Normative feedback [90]	X			X
Social cognitive theory [91]	X	X	X	X
Structured relapse prevention [92]	X			
Targeting and tailoring [93]		X	X	
Transtheoretical model [94]				X

Table 2 outlines intervention launch dates, data acquisition dates, number of registrants, number of actors, total posts, and number of subjects used in analysis from their intervention DHSN inception until December 31, 2015.

**Table 2:** Four social networks.

Social network	Social network launch date	Data acquisition date	Number of days active	Number of subjects registered in program	Number of actors, n (%)	Number of actor posts^a^	Number of subjects in analysis, n (%)^a^
Problem drinking	Dec 26, 2005	Dec 31, 2015	3658	5049	1085 (21.49)	16,231	4784 (94.75)
Depressive disorder	Feb 6, 2003	Dec 31, 2015	4712	11,675	2065 (17.69)	20,516	1958 (16.77)
Panic disorder	January 23, 2002	Dec 31, 2015	591	9783	3579 (36.58)	61,743	6151 (62.87)
Smoking cessation	Sep 26, 2001	Dec 31, 2015	5210	52,396	8452 (16.13)	521,997	12,061 (23.01)
Total	n/a^b^	n/a	18,671	78,903	15,181 (19.24)	620,487	25,178 (31.91)
Mean	n/a	n/a	4688	19,726	3795 (19.24)	155,122	6239 (31.63)

^a^Moderator posts removed.

^b^n/a: not applicable.

### Data Collected at Registration

Demographic characteristics (age, gender, highest level of education obtained, current occupation), and indication-specific details (Table 3) were collected at registration. Program registration and participation were free; however, consenting to the use of personal data for research purposes was a requirement.

**Table 3:** Indication-specific data collected at registration.

Intervention	Indication-specific data	Measurement
**Problem drinking**	Average drinks per day	Drop-down menu 0-30+
	Program goal: cut down, stop, unsure	Likert scale
**Depressive disorder**	Depression rating over past 2 weeks	Likert scale 0-10
	Level of distress over past 2 weeks	Likert scale 0-10
	Level of interference over past 2 weeks	Likert scale 0-10
	Tried cognitive behavior therapy in the past	Yes or no
	Currently being treated	Yes or no
	Using program with health care professional	Yes or no
**Panic disorder**	Number of attacks over past 2 weeks	Drop-down menu 0-51+
	Average fear rating during attack	Likert scale 0-10
	Attack interference with average daily life	Drop-down menu 0-4
	Attack causing avoidance	Drop-down menu 0-4
	Tried cognitive behavior therapy in the past	Yes/No
	Use of program with health care professional	Yes/No
**Smoking cessation**	Smoking patterns: ≥ 1 cigarette per day, occasional smoker, recently quit	Drop-down menu
	Last cigarette: >24 hours, <24 hours	Radio button
	Cigarettes per day	Drop-down menu 0-100+
	Total years smoked	Drop-down menu 0-75+
	Minutes to first cigarette: >60, 31-60, 6-31, ≤5	Drop-down menu
	Past year quit attempts > 24 hours	Drop-down menu 0-10+
	Number of cohabitant smokers	Drop-down menu 0-10+
	Fagerstrom dependency score (very low, low, moderate, high, very high)	Internal calculation

### Objective

As a first step in profiling actors based on characteristics, and to investigate the feasibility of developing predictive models that identify superusers in real time, the objective of this study was to examine the association between number of posts and actor demographic and indication-specific variables inputted at registration.

## Methods

### Sample

Data were extracted from the custom SQL DHSN databases of the 4 digital health interventions. As they contained full data sets, samples totaling 24,954 registrants and 3285 actors were used in the analysis (Table 4).

**Table 4:** Sample size.

Intervention	Sample size	Sample size actors	Sample size posts
Problem drinking	4484	884	12,914
Depressive disorder	1958	206	3190
Panic disorder	6151	585	18,921
Smoking cessation	12,061	1610	90,894
Total sample	24,954	3285	125,919

### Regression Models

A total of 5 models were developed to explore whether posting behavior was associated with demographics characteristics and indication-specific severity amongst all registrants (Table 5).

**Table 5:** Regression models for all subjects.

Model	Equation
1	*ProblemDrinkingPostsAllRegistrants* = β_0_+ β_1_ *Age *+ β_2_ *Gender* + β_3_*Education* + β_4_ *Occupation* + *β*_5_*DrinksPerDay* + β_6_*Goal* + ϵ
2	*DepressiveDisorderPostsAllRegistrants* = β_0_+ β_1_*Age* + β_2_*Gender* + β_3_*Education* + β_4_*Occupation* + β_5_*Rating* + β_6_*Distress* + β_7_*Interference* + β_8_*CBT* + β_9_*Treated* + β_10_*Professional* + ϵ
3	*PanicDisorderPostsAllRegistrants* = β_0_+ β_1_*Age* + β_2_*Gender* + β_3_*Education* + β_4_*Occupation* + β_5_*Attacks* + β_6_*Fear* + β_7_*Interference* + β_8_*Avoidance* + β_9_*CBT* + β_10_*Professional* + ϵ
4	*SmokingCessationPostsAllRegistrants* = β_0_+ β_1_*Age* + β_2_*Gender* + β_3_*Education* + β_4_*Occupation* + β_5_*Patterns* + *β*_6_*LastCigarette* + *β*_7_*CigarettesPerDay* + β_8_*YearsSmoked* + β_9_*FirstCigarette*+ β_10_*PastQuits* + β_11_*CohabitantSmokers* + β_12_*FagerstromScore* + ϵ
5	*TotalPostsAllRegistrants* = β_0_+ β_1_*Age* + β_2_*Gender* + β_3_*Education* + β_4_*Occupation* + ϵ

Another 5 additional regression models were developed to explore whether posting behavior was associated with demographics characteristics and indication-severity amongst actors (Table 6).

**Table 6:** Regression models for actors.

Model	Equation
6	*ProblemDrinkingPostsActors* = β_0_+ β_1_*Age* + β_2_*Gender* + β_3_*Education* + β_4_*Occupation* + β_5_*DrinksperDay* + β_6_*Goal* + ϵ
7	*DepressiveDisorderPostsActors* = β_0_+ β_1_*Age* + β_2_*Gender* + β_3_*Education* + β_4_*Occupation* + β_5_*Rating* + β_6_*Distress* + β_7_*Interference* + β_8_*CBT* + β_9_*Treated* + β_10_*Professional* + ϵ	
8	*PanicDisorderPostsActors* = β_0_+ β_1_*Age* + β_2_*Gender* + β_3_*Education* + β_4_*Occupation* + β_5_*Attacks* + β_6_*Fear* + β_7_*Interference* + β_8_*Avoidance* + β_9_*CBT* + β_10_*Professional* + ϵ
9	*SmokingCessationPostsActors* = β_0_+ β_1_*Age* + β_2_*Gender* + β_3_*Education* + β_4_*Occupation* + β_5_*Patterns* + β_6_*Last Cigarette* + β_7_*CigarettesPerDay* + β_8_*YearsSmoked* + β_9_*FirstCigarette* + β_10_*PastQuits* + β_11_*CohabitantSmokers* + β_12_*FagerstromScore* + ϵ	
10	*TotalPostsActors* = β_0_+ β_1_*Age* + β_2_*Gender* + β_3_*Education* + β_4_*Occupation* + ϵ

Dummy variables were created for categorical data, with 1 dummy variable excluded during regressions. Analyses were performed with Stata version 13 (Stata Corp LLP, College Station, TX, USA).

As outlined in previous research conducted on the 4 DHSNs, the number of posts per actor is right skewed, indicating the presence of a power law [44]. Negative binomial regression was employed as the method of analysis for 3 reasons. First, the dependent variable in our model, number of observations, is counted as integers only. Second, negative binomial regression can capture the skewness of the data. Third, Poisson distribution requires the mean and the variance of the model to be identical and in each of the models, the hypothesis of equidispersion is rejected.

### Ethics

All data collection policies and procedures adhered to international privacy guidelines [95-97] and were in accordance with the Helsinki Declaration of 1975, as revised in 2008 [98]. The study was consistent with the University Research Ethics Committee procedures at Henley Business School, University of Reading, and was exempt from full review.

## Results

### R-Squared Values

All 5 models had low R^2^ values (see Table 7 and Multimedia Appendix 1).

### Regression Analysis: Demographic Variables

A total of 4 independent demographic variables were included in each of the 10 models (Table 8).

In 9 of the models, age was positively and significantly associated with number of posts (beta range =.13-.4). This means that as age of registrants increased, number of posts increased marginally.

Education was positively and significantly associated to the number of posts in 6 models (beta range =.082-.315). This means that within these 6 models, number of posts increases by less than 1 with every unit increase in education category.

Gender was negatively and significantly associated number of posts in 4 models (beta range =−.766 to −.272). This means that within these 4 models, number of posts decreased by less than 1 with male registrants.

Registrants had the option of selecting from 1 of 12 occupations. Compared with registrants who indicated that they were full-time students, occupation was positively associated with number of posts in 14 cases (beta range =.377-5.301), and negatively associated with number of posts in 19 cases (beta range =-2.609 to -.587).

The variable *occupation not listed* was selected with the greatest frequency 60% (6/10), and was positively and significantly associated to the number of posts in 4 of these 6 models (beta range =.488-.703), but negatively and significantly associated to the number of posts in 2 of these 6 models (beta range =−1.314 to −.945).

**Table 7:** R^2^ values for ten models.

Model	1	2	3	4	5	6	7	8	9	10
R^2^	0.016	0.013	0.02	0.043	0.026	0.027	0.018	0.061	0.086	0.031

**Table 8:** Statistically significant demographic independent variables (all models).

Independent variable		Model 1 beta (*P* value)	Model 2 beta (*P* value)	Model 3 beta (*P* value)	Model 4 beta (*P* value)	Model 5 beta (*P* value)	Model 6 beta (*P* value)	Model 7 beta (*P* value)	Model 8 beta (*P* value)	Model 9 beta (*P* value)	Model 10 beta (*P* value)	Percentage significant
Gender		−.272 (.001)	−.766 (<.001)		−.422 (.03)	−.365 (.005)						40
Age		.400 (<.001)	.234 (<.001)		.324 (<.001)	.130 (.009)	.322 (<.001)	.136 (<.001)	.138 (.012)	.285 (<.001)	.184 (<.001)	90
Education		.146 (<.001)			.315 (.001)	.195 (.001)	.082 (.002)	.095 (.01)			.139 (.008)	60
**Occupation**												
	Full-time student (reference)											
	Stay at home mom or dad				−.720 (.04)					−1.057 (<.001)		20
	Management	.546 (.004)							−1.675 (.002)			20
	Teacher or professor			−2.348 (.005)	−1.139 (.01)	.810 (.02)			−2.609 (.000)	−.949 (.02)		50
	Administrative, financial or clerical sales or service	.519 (.001)					.377 (.01)	.852 (.005)	-.894 (.035)			40
	Technologist or technical occupation	.532 (.003)										10
	Farming, forestry, fishing or mining	1.016 (<.001)			5.301 (<.001)		.400 (.04)			3.793 (<.001)		30
	Trades, transport or equipment operator				−1.564 (.02)	−1.047 (.007)	−.690 (<.001)				−.696 (.05)	40
	Processing, manufacturing or utilities					−.846 (.02)	−.641 (.001)					20
	Unemployed at present or on work leave	.479 (.008)							−.820 (.02)	−.587 (.02)		20
	Professional services (eg, certified accountant, lawyer, doctor)				−.856 (<.001)							10
	Occupation not listed	.703 (<.001)				.825 (.001)	.488 (.001)		−1.314 (.002)	−.945 (.001)	.647 (.004)	60

### Regression Analysis: Indication-Specific Variables

In total, 10 indication-specific variables were tested for their association with posting behavior in the 2 addiction health interventions (Table 9).

### Problem Drinking Intervention

In the problem drinking intervention, registrants had the option of selecting 1 of the 3 program goals. Compared with registrants who indicated that they wanted to cut down, *quit drinking* was positively and significantly associated with the number of posts in model 2 (beta=.463, *P*=.02). The option *not sure* was negatively and significantly associated with the number of posts in model 2 (beta=−. 460, *P*=.02) and model 7 (beta=−.509, *P=*.001).

**Table 9:** Statistically significant indication-specific independent variables (addiction interventions).

Independent Variables		Model 2 beta (*P* value)	Model 7 beta (*P* value)	Model 5 beta (*P* value)	Model 10 beta (*P* value)
**Goal**					
	Cut down (reference)			n/a^a^	n/a
	Quit drinking	.463 (.02)		n/a	n/a
	Not sure	−.460 (.02)	−0.509 (.001)	n/a	n/a
**Smoking patterns**					
	≥ one cigarette per day, occasional smoker, recently quit	n/a	n/a	.278 (.001)	
	Last cigarette: >24 hours, <24 hours	n/a	n/a	.534 (.002)	
	Cigarettes per day	n/a	n/a		
	Total years smoked	n/a	n/a	.040 (<.001)	.025 (.001)
	Minutes to first cigarette: >60, 31-60, 6-31, ≤5	n/a	n/a	.705 (<.001)	.625 (<.001)
	Past year quit attempts > 24 hours	n/a	n/a	−.048 (.02)	−.054 (.001)
	Number of cohabitant smokers	n/a	n/a		
	Fagerstrom dependency score (very low, low, moderate, high, very high)	n/a	n/a	0.657 (.001)	0.651 (<.001)

^a^n/a: not applicable.

### Smoking Cessation Intervention

In model 5, increased cigarette consumption (smoking patterns) (beta=.278, *P*=.001) and having a cigarette within the past 24 hours (last cigarette) were positively and significantly associated with posting behavior (beta=.534, *P*=.002).

In both models, increases in total years smoked (beta=.040, *P*<.001; beta=.025, *P*=.001), decreases in minutes to first cigarette (beta=.705, *P*=.002; beta=.625, *P*<.001), and higher Fagerstrom dependency scores (beta=.657, *P*=.001; beta=.651, *P*<.001) were positively and significantly associated with posting behavior. Having a greater number of quit attempts was negatively and significantly associated with posting (beta = **−**.048, *P*=.02; **−**.054, *P*=.001).

### Regression Analysis: Indication-Specific Variables in Two Mental Health Interventions

Ten indication-specific variables were tested for their association with posting behavior in the 2 mental health interventions. Whether a participant had *tried cognitive behavior therapy in the past* and was *using of the program with a health care* professional were asked in both mental health interventions (Table 10).

#### Past Cognitive Behavior Therapy Experience

In models 3, 4, and 9 posting behavior was positively and significantly associated with experience with CBT (beta= .851, *P*=.01; beta=1.118, *P*<.001; beta=.870, *P*<.001).

**Table 10:** Statistically significant indication-specific independent variables (mental health).

Independent variables	Model 3 beta (*P* value)	Model 8 beta (*P* value)	Model 4 beta (*P* value)	Model 9 beta (*P* value)
Depression rating past 2 weeks (0-10)			n/a^a^	n/a
Level of distress past 2 weeks (0-10)			n/a	n/a
Level of interference past 2 weeks (0-10)			n/a	n/a
Currently being treated			n/a	n/a
Tried cognitive behavior therapy in the past	.851 (.01)		1.118 (<.001)	.870 (<.001)
Number of attacks over past 2 weeks Using program with a health care professional	n/a	n/a	.054 (.03)	
Average fear rating during attack	n/a	n/a		−.099 (.01)
Attack interference in average daily life	n/a	n/a	.406 (<.001)	.224 (.01)
Attack causing avoidance	n/a	n/a		
				

^a^n/a: not applicable.

### Depression Intervention

In the depression interventions, other than past CBT experience, there were no statistically significant associations with posting behavior.

### Panic Disorder Intervention

In the panic disorder intervention, attacks interfering in average daily life were positively and significantly associated with posting behavior (beta=.406, *P*<.001; beta=.224, *P*=.01). In model 4, increases in number of attacks over the past 2 weeks were positively and significantly associated with posting (beta=.054, *P*=.03), and in model 9 average fear rating during an attack was negatively and significantly associated with posting (beta=−.099, *P*=.01).

## Discussion

### Principal Findings

Despite observable statistically significant results in demographic and indication-specific data, all regressions had low R^2^ values, and their impact on superuser behavior was minimal. As mentioned previously, all models fail to explain the variance of the dependent variables.

Based on the results in 4 of the 10 models, females tend to post more than males. However, these results should be interpreted with caution as the impact was minimal (beta range=−.766 to −.272) and only statistically significant in all subject models. These results also do not confirm the gender of superusers.

Increased posting with age was positively and statistically significant in 9 of the 10 models, although the increase is negligible and should be interpreted with caution (beta range=.130-.400). For example, the analysis did not consider whether addiction treatment for smoking cessation, or if treatment for mental health issues, also coincides with age.

Although the impact is minimal, increased education was related to increases in posting behavior in 6 of the 10 models (beta range=.082-.315). The issue of education level and use of medical resources has a rich history in the literature and is nonconclusive. For example, one might assume that actors with higher levels should have better knowledge seeking skills and make limited use of DHSNs, or conversely, that actors with lower education levels and fewer formal resources would use DHSNs with greater intensity.

A recent qualitative review on factors affecting therapeutic compliance found the effect of education level to be equivocal [99]. While some studies found that patients with higher levels of education might have higher compliance, others found that patients with lower levels of education or no formal education were more compliant. The authors concluded that education level was not a good predictor of therapeutic compliance, and our findings reflect this in regards to education being associated with posting.

In the smoking cessation intervention, inexperienced quitters who have smoked longer, have increased dependency, and have recently quit, tend to post more. This supports past research indicating that the intervention’s DHSN primarily acts as a relapse prevention tool for new quitters [45,82]. If this finding is true it highlights the importance of detecting and supporting superusers as they primarily respond to, and support, new users.

It was interesting to note that *experience with cognitive behavior therapy* was associated with posting behavior in 3 of the 4 mental health models, though this impact was minimal (beta range=.851-1.118).

### Future Research

The results of this study suggest that demographic or indication-specific variables have limited association with the creation of externalities in DHSNs. What, if anything, may be associated with posting behavior? If superusers are key to the growth and sustainability of DHSNs, how can they be detected?

The real-time assessment of phenotype, or observable traits resulting from the interaction of an individual in an environment, have recently been recognized as key to the next frontier of medicine [100]. Phenotypes differ from demographic and indication-specific data as they give insight on behavior. Although traditionally difficult to detect, some phenotypes are now being recognized through big data analysis.

For example, a recent study identified the ability to use natural language processing to detect phenotypes in electronic health records [101]. Another study found that an individual’s personal attitudes including use of addictive substances, happiness, and sexual orientation can be detected through Facebook likes [102], and Instagram photos and Twitter feeds have been shown to contain predictive markers of depression [103,104].

DHSN content may contain rich sources of phenotypes as an post or an actor’s profile may include avatars, images, badges or awards for participation, likes or other semiotic indicators of support from other members, or links to specific outside resources. Post content may be mined for specific keywords, phrases, or even tone. Time of post, time between posts, response to specific types of content or members, or other time-based interactions may also be indicative of specific behavior. Recent health care informatics research has also identified a relationship between increased systems use and outcomes, and a variety of unique system measures that may help categorize behaviors [105].

A challenge is that even if phenotypes can be predicted, risk-stratifying behavior may prove difficult. However, the medication adherence literature, which generally classifies patients as full compliers, partial compliers, or noncompliers may give insights on categorizing behavior similar to nonadherence [106] and research is beginning to investigate indication-specific factors that categorize patients and their motivations [107-110]. Future research into adherence to DHSNs might also consider the feasibility of stratifying actors according to real-time behavior.

In some respects, the low R^2^ values in the models and lack of statistically significant variables in this study expose the limitations of big data. Popular belief holds that large data sets of survey data will contain insights and intelligence that have been previously unobtainable [111-113], and the promise of big data is so compelling that laymen are being encouraged to experiment with sophisticated techniques that previously required a high degree of training [114]. Whereas increased knowledge and interdisciplinary training and collaboration are certainly positive, as in this study, results from the analysis of large datasets pertaining to specific demographic characteristics or indication-specific variables may, at best, illustrate the complexity of predicting human behavior.

### Strengths and Limitations

The results of this study are from “real world” social networks and the main strengths are the longevity of the DHSNs, the number of posts, the 4 separate indications, and that 2 of the social networks in the study were focused on mental health, and the remaining 2 on addictions.

Ideally, data from this study would be derived from a randomized controlled experiment. However, it would be difficult, if not impossible, to recruit a study population and execute a study in a similar sample. We are not aware of any other study in the health care literature with such an extensive and complete dataset, and as such, results should be interpreted accordingly.

A strength and limitation is that the populations analyzed are self-selecting populations that actively sought help. In the context of this study it was helpful to have datasets of active and engaged participants. However, these results may not be indicative of populations of patients in health plans, hospital networks, or mass public health campaigns.

A limitation to this study is that demographic and indication-specific data was self-report. Self-report data is common in digital health studies, and the consensus is that data from subjects is at least as reliable as pencil-and-paper questionnaires [115-122]. However, due to the anonymous nature and nonrandomization of study subjects, results should be interpreted with caution.

### Conclusions

Based on the large number of study participants, variation in DHSN theme, and extensive time-period, we did not find strong evidence that demographic characteristics or indication severity sufficiently explain the variability in number of posts per actor. Researchers should investigate alternative methods and models that may identify individuals who promote DHSN growth.

## Appendix

Multimedia Appendix 1

## Abbreviations

DHSN: Digital health social network
