# Supplementary material for: Demographic and Indication-Specific Characteristics Have Limited Association With Social Network Engagement: Evidence From 24,954 Members of Four Health Care Support Groups
Source: J Med Internet Res. 2017 Feb 17;19(2):e40. doi: 10.2196/jmir.6330 (PMC5336601; doi:10.2196/jmir.6330)

## Slide 1
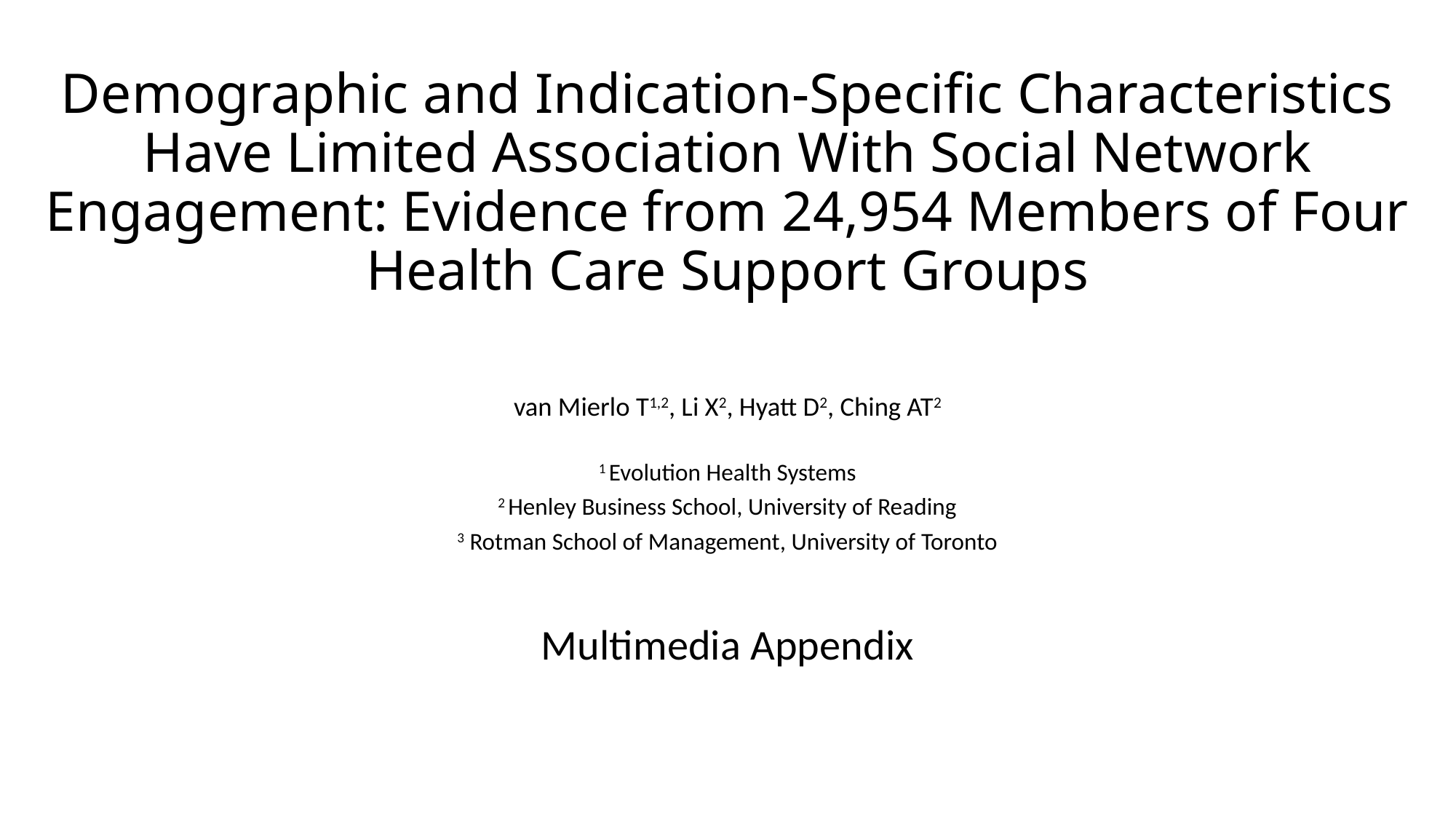

# Demographic and Indication-Specific Characteristics Have Limited Association With Social Network Engagement: Evidence from 24,954 Members of Four Health Care Support Groups
van Mierlo T1,2, Li X2, Hyatt D2, Ching AT2
1 Evolution Health Systems
2 Henley Business School, University of Reading
3 Rotman School of Management, University of Toronto
Multimedia Appendix

## Slide 2
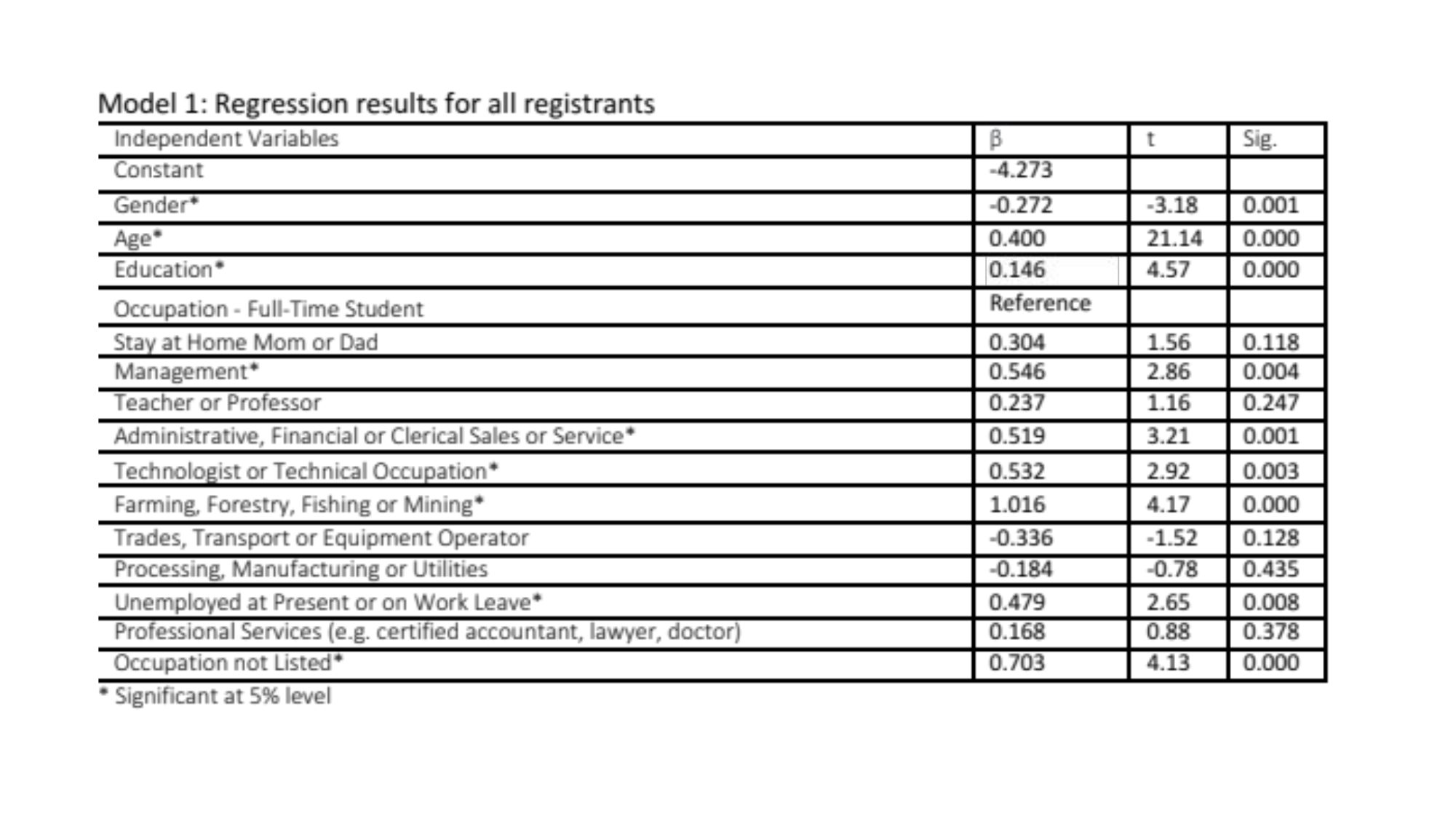

## Slide 3
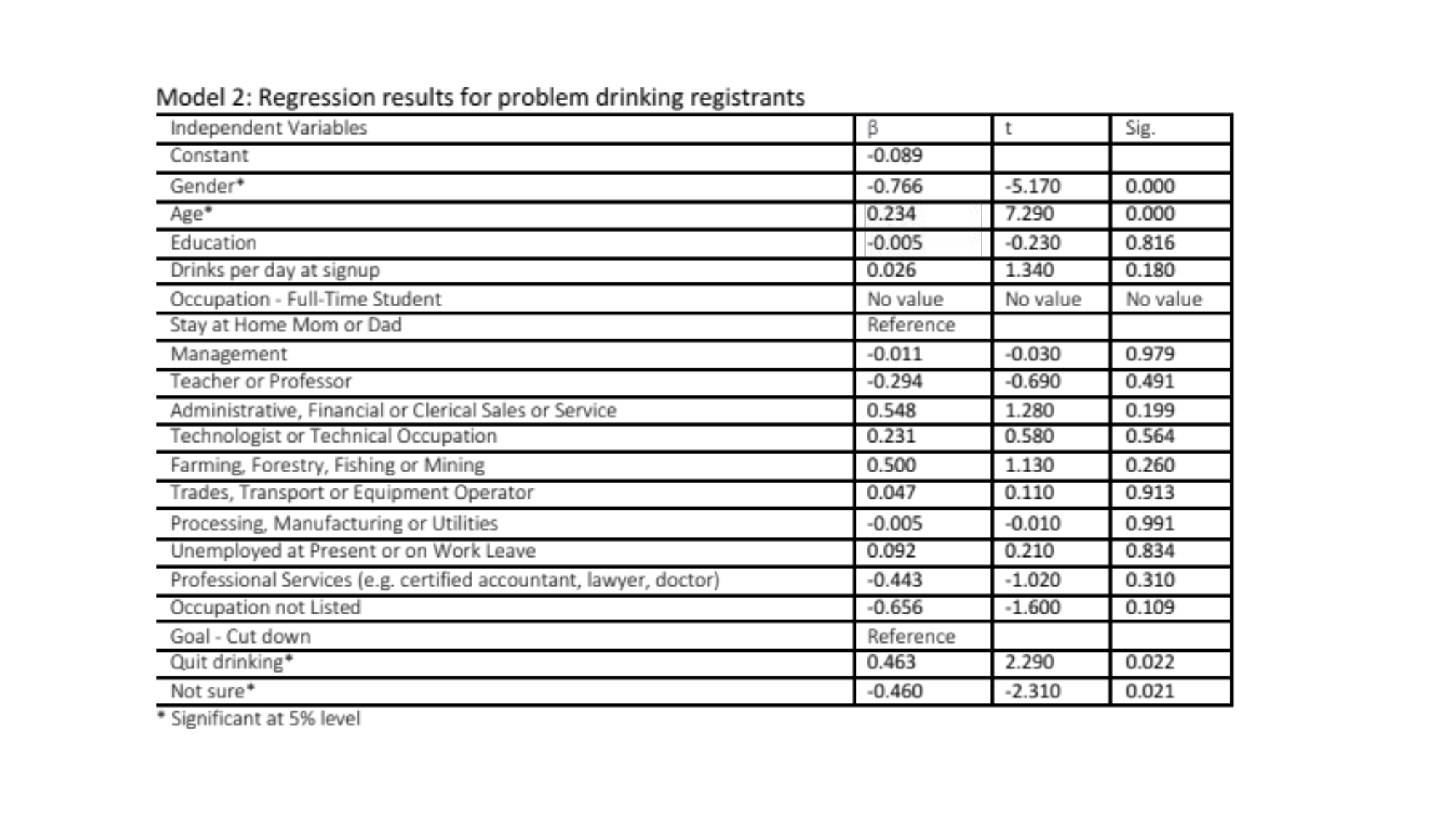

## Slide 4
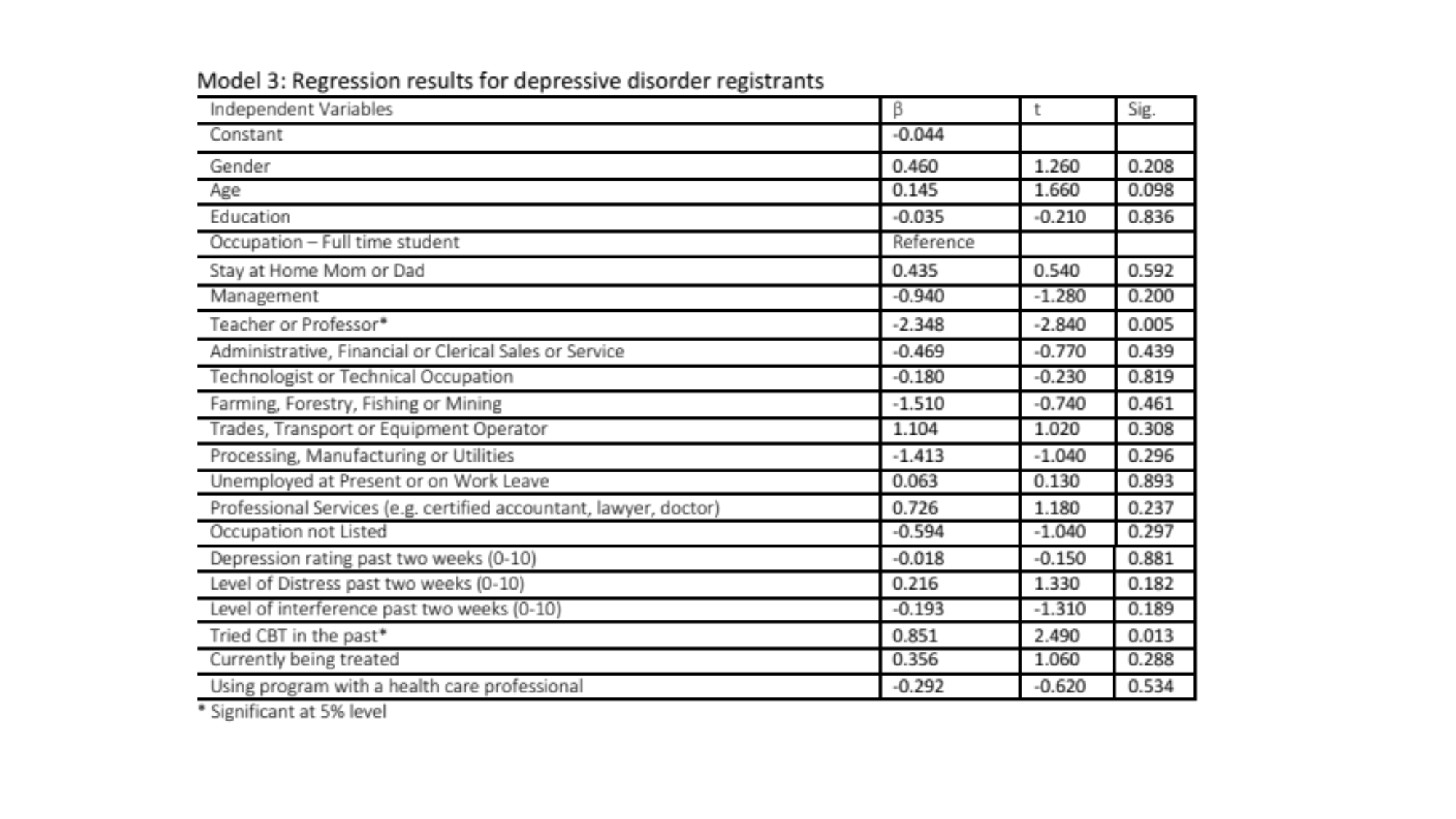

## Slide 5
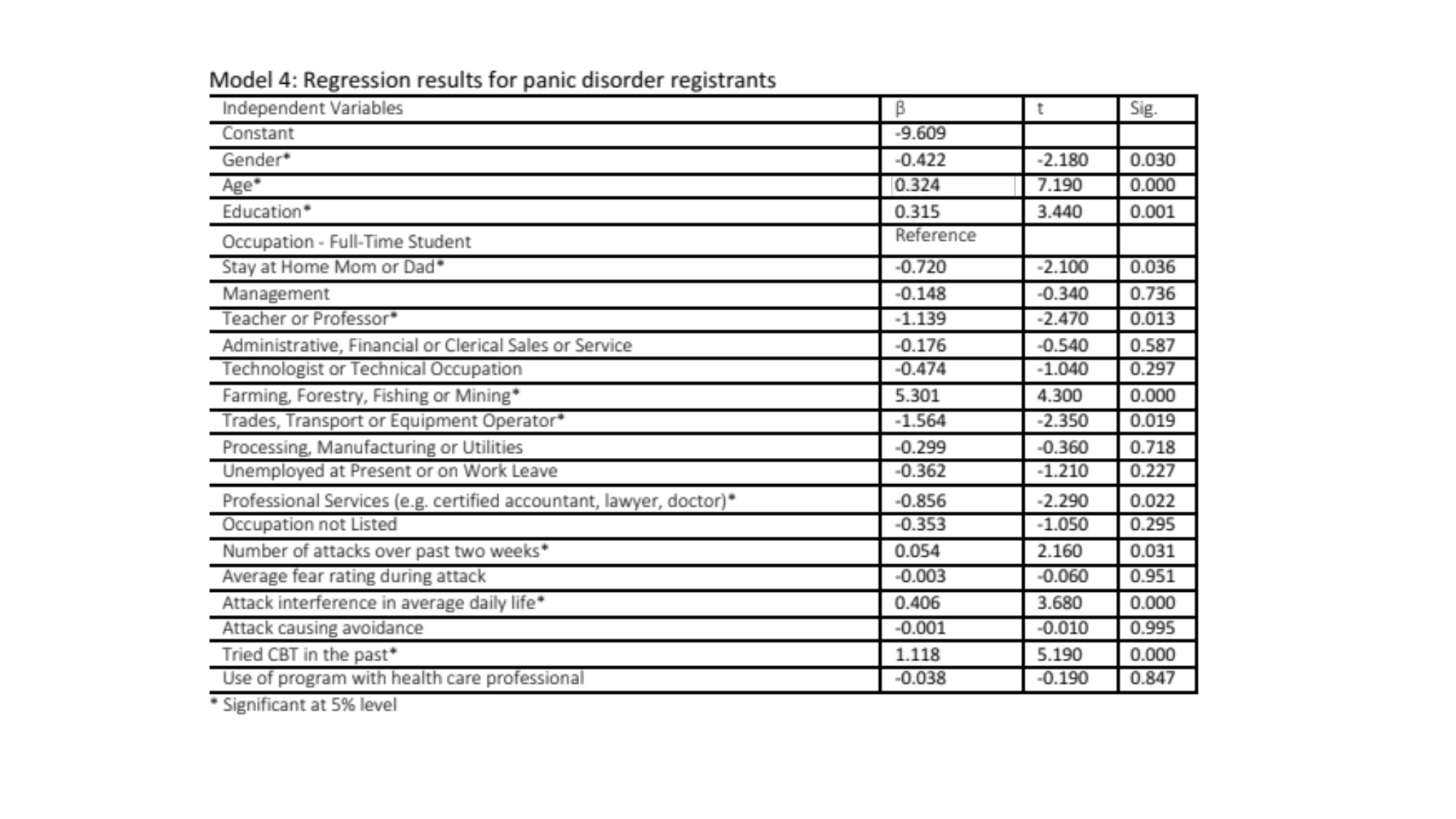

## Slide 6
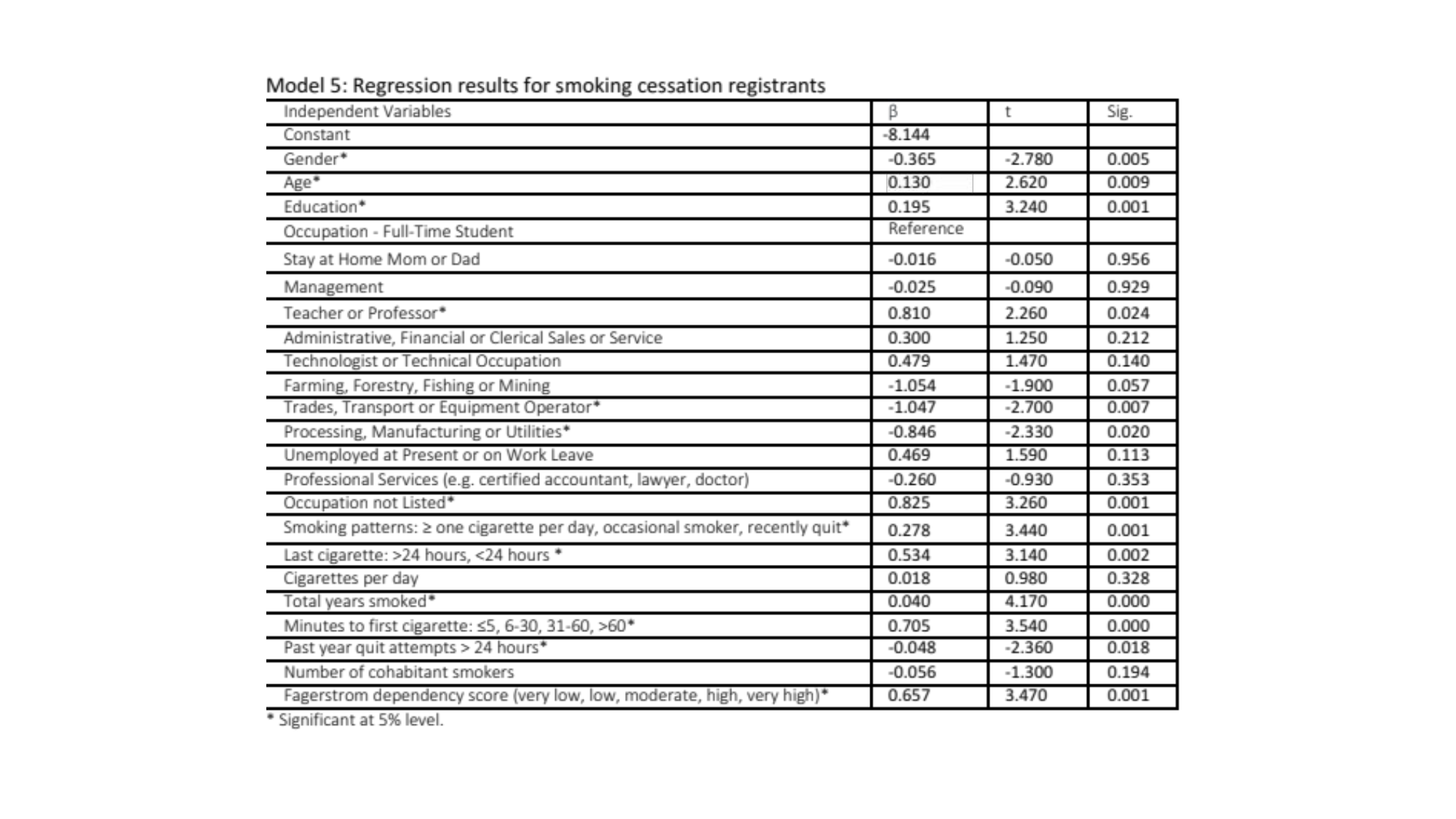

## Slide 7
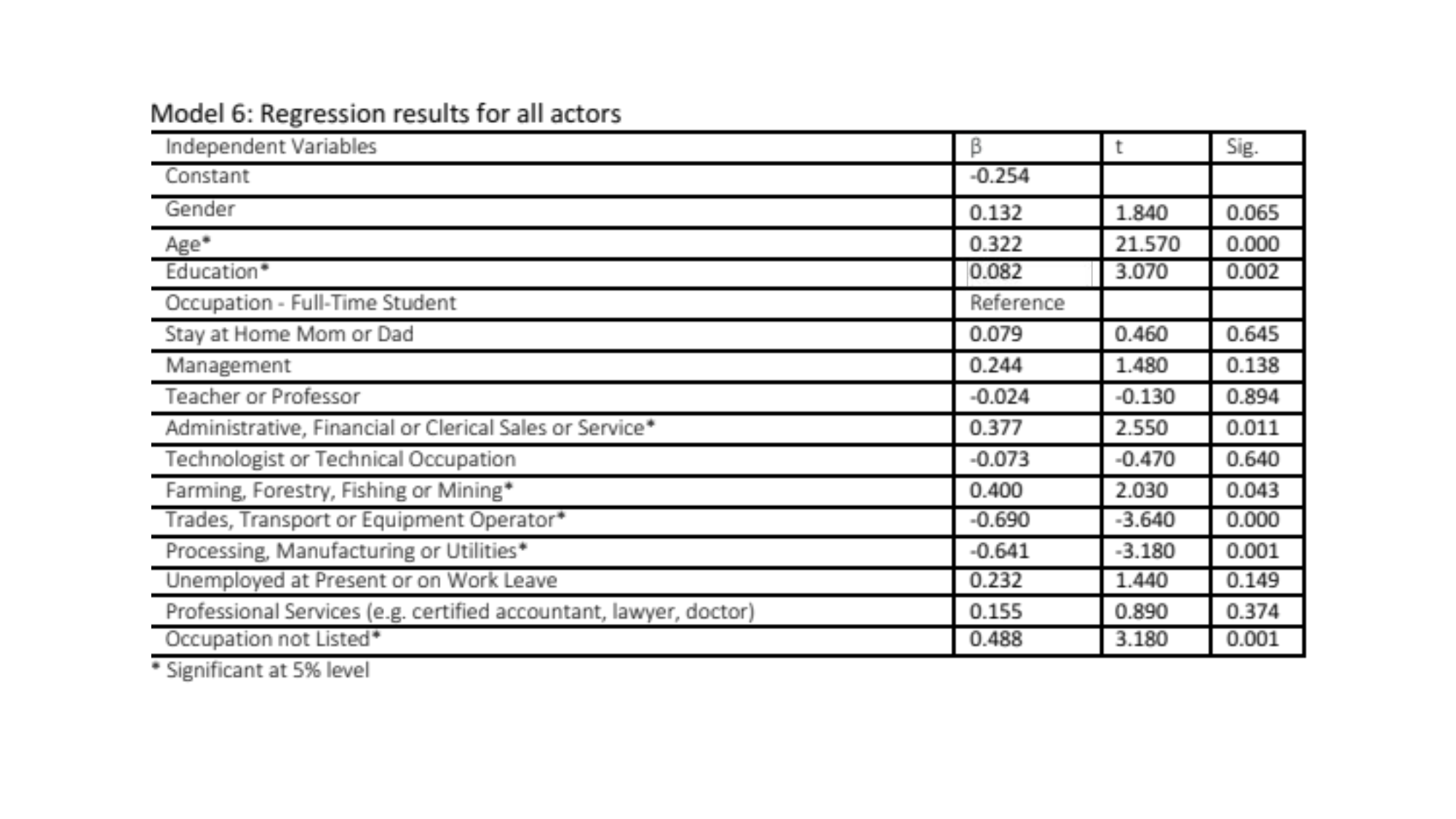

## Slide 8
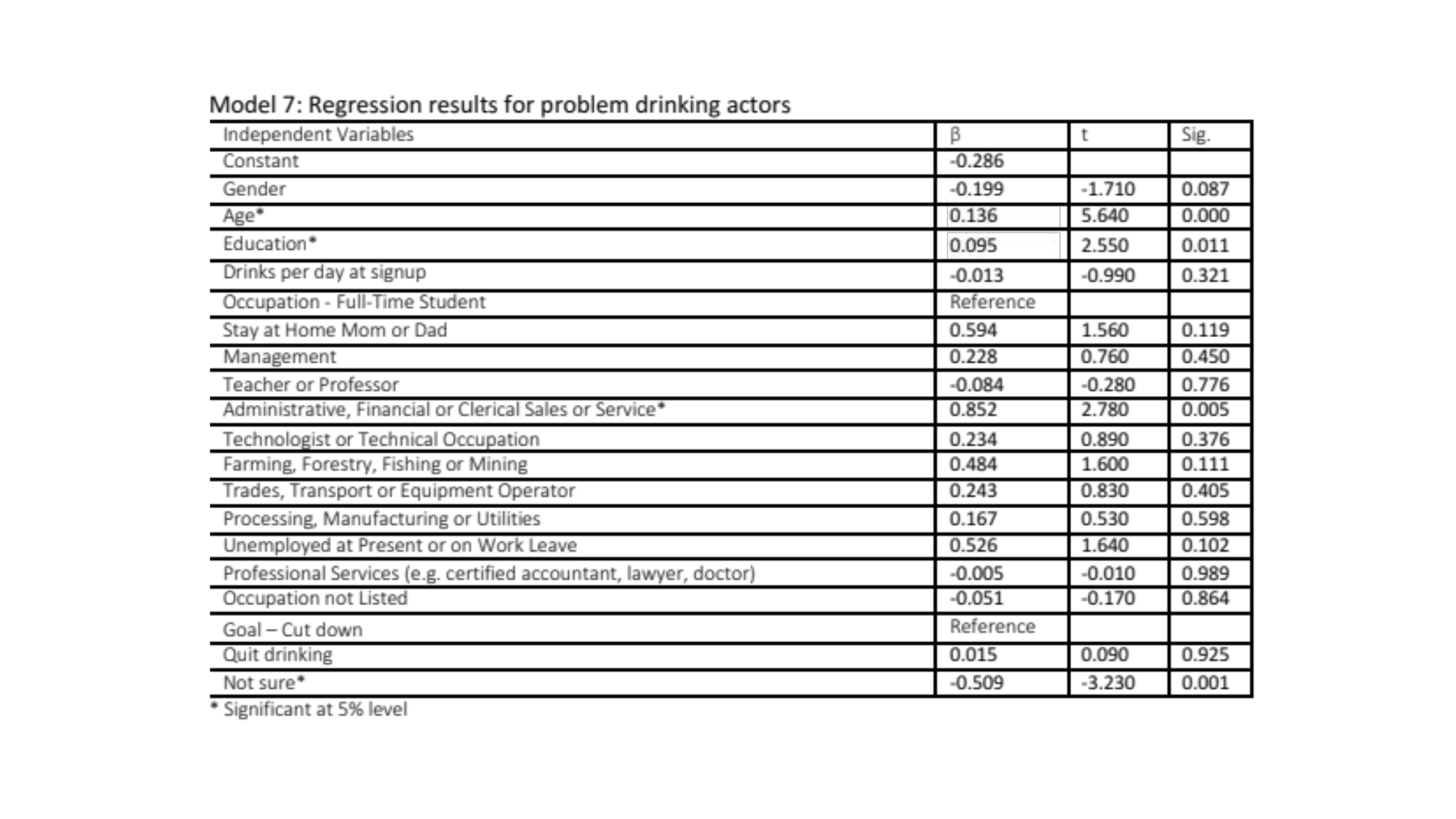

## Slide 9
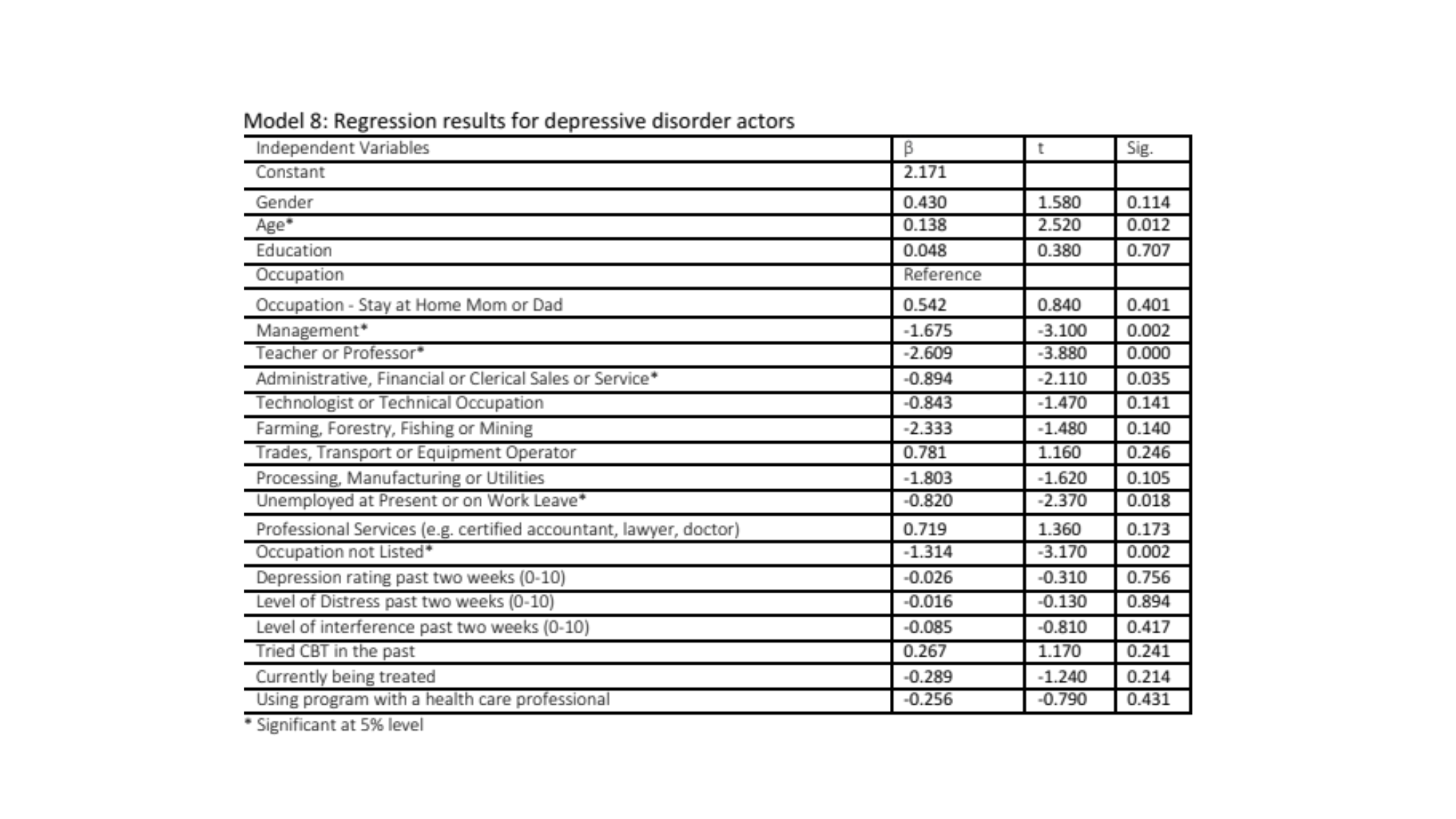

## Slide 10
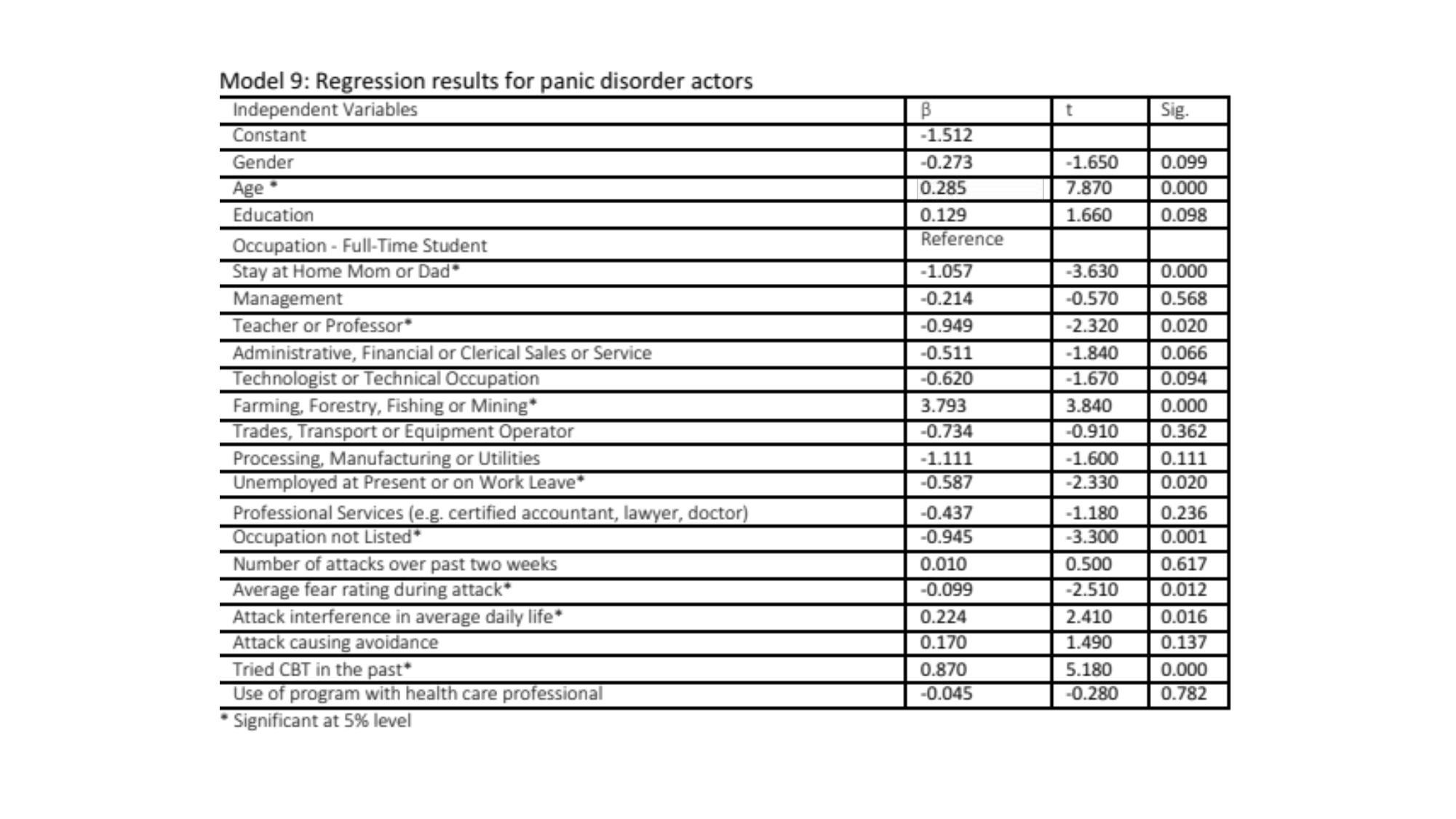

## Slide 11
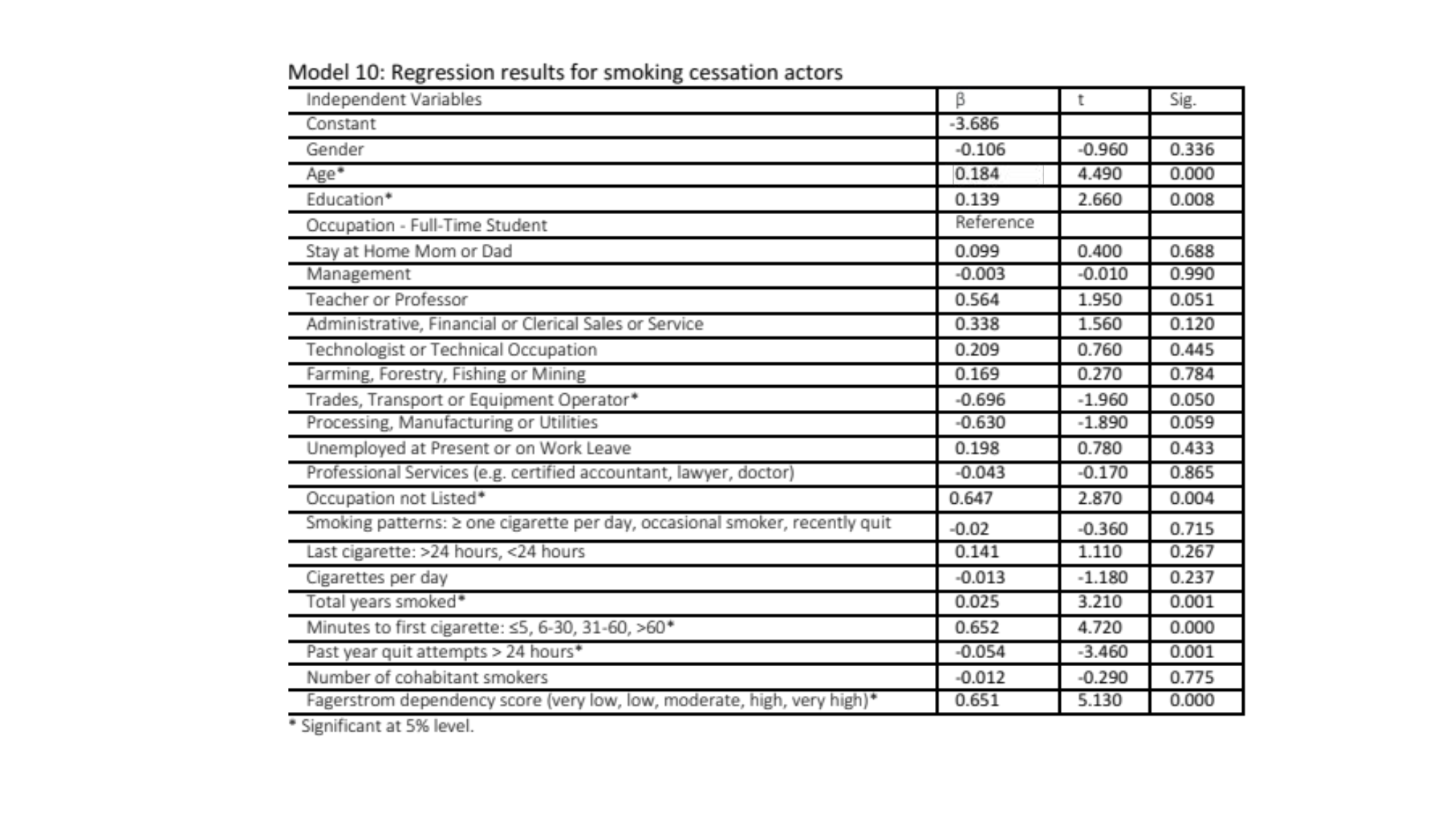

Supplement: Multimedia Appendix 1 [file jmir_v19i2e40_app1.pptx]
